# Serum Levels of Soluble IL-2R, CD4 and CD8 in Chronic Active HCV Positive Hepatitis

**DOI:** 10.1155/S0962935194000256

**Published:** 1994

**Authors:** C. Caruso, G. Candore, D. Cigna, S. Tripi, G. Di Gaetano, G. Migneco, G. Montalto, I. Ruggieri, A. Notarbartolo

**Affiliations:** 1 Istituto di Patologia generale Università di Palermo Corso Tukory 211 Palermo 90134 Italy; 2 Istituto di Clinica Medica e Terapia Medica Università di Palermo Via del Vespro Palermo 90100 Italy; 3 Istituto di Medicina Interna e Geriatria Università di Palermo Via del Vespro Palermo 90100 Italy

## Abstract

The aim of the present study was to compare serum levels of soluble forms of interleukin-2 receptor, CD4 and CD8, released by lymphocytes during activation ofthe immune system, in patients with histologically verified chronic active hepatitis associated to hepatitis C virus infection, with those in healthy subjects. Significantly higher levels of soluble IL-2R and soluble CD8 were found in patients with chronic active hepatitis compared with controls. In contrast no difference was found for soluble CD4 values in the two groups. No correlations were found for both sIL-2R and sCD8 and these two molecules with other parameters of liver function. These results indicate that in these patients there is a general activation of the immune system, but the lack of correlation with parameters of liver function strengthens the suggestion that this activation does not play a role in the pathogenesis of chronic type C hepatitis.


Research Paper

Mediators of Inflammation 3, 185–187 (1994)

### Serum levels of soluble IL-2R, CD4 and CD8 in chronic active HCV positive hepatitis

C. Caruso,[1,CA] G. Candore,[1] D. Cigna,[1] S. Tripi,[2] G. Di Gaetano,[2] G. Migneco,[2] G. Montalto,[3] I. Ruggieri[3] and A. Notarbartolo[3]

[1]Istituto di Patologia generale, Università di Palermo, Corso Tukory 211, 90134 Palermo, Italy; [2]Istituto di Clinica Medica e Terapia Medica; [3]Istituto di Medicina Interna e Geriatria, Università di Palermo, Via del Vespro, 90100 Palermo, Italy

[CA] Corresponding Author

**The aim of the present study was to compare serum levels of soluble forms of interleukin-2 receptor, CD4 and CD8, released by lymphocytes during activation of the immune system, in patients with histologically verified chronic active hepatitis associated to hepatitis C virus infection, with those in healthy subjects. Significantly higher levels of soluble IL-2R and soluble CD8 were found in patients with chronic active hepatitis compared with controls. In contrast no difference was found for soluble CD4 values in the two groups. No correlations were found for both sIL-2R and sCD8 and these two molecules with other parameters of liver function. These results indicate that in these patients there is a general activation of the immune system, but the lack of correlation with parameters of liver function strengthens the suggestion that this activation does not play a role in the pathogenesis of chronic type C hepatitis.**

**Key words**: Chronic liver diseases, HCV, sCD4, sCD8, sIL-2R

### Introduction

During activation, lymphocytes secrete glycoproteins related to their surface proteins, including interleukin-2 receptor (IL-2R), CD4 and CD8 soluble forms. These glycoproteins are found in relatively large amounts in the circulation and it is possible to obtain normal ranges for normal persons, with upper limits. This allows the detection of states of increased production of soluble interleukin-2 receptor (sIL-2R), soluble CD4 and CD8 (sCD4 and sCD8) corresponding to increased activation of the immune system. Increased levels of a soluble form of these glycoproteins have been observed in the serum of patients with malignant, autoimmune and allergic disorders, as well as in subjects affected by systemic infectious diseases or undergoing allograft rejection. Thus, the release of these soluble molecules appears to be a characteristic marker of lymphocyte or mononuclear cell (MNC) activation. In infectious diseases the elevation of serum soluble molecules is likely to indicate immune system activation in response to antigenic challenge by infectious agents. Particularly, levels of sCD8 are increased in diseases where there is a prominent role of cytotoxic T cells, such as viral infection.[1–6]

It has been suggested that hepatitis C virus (HCV) is directly cytopathic and liver cell damage in HCV infection is not mediated by cytotoxic T cells directed against the viral determinants expressed on the infected hepatocytes. Nevertheless, several results suggest that in these patients there may be various impairments of immune functions, but no relationship has been demonstrated between the impairment and the natural history of the disease.[7–10] In the present paper, to gain insight into the significance of these impairments, serum levels of sIL-2R, sCD4 and sCD8 in patients with chronic HCV infection have been studied.

### Materials and Methods

*Sample population*: A total of 84 subjects was studied; 33 were patients with histologically verified chronic type C hepatitis (twelve females and 21 males, range 40–65 years). No other aetiological factors were associated, such as positivity for HBc antibodies, autoimmune markers, alcohol consumption, Wilson's disease, $\alpha_1$ anti-trypsin deficiency. Transfusion or previous surgery were the main risk factors in most patients.

The remainder consisted of 51 healthy adults (26 females and 25 males, range 20–64 years). None of these subjects had a history of prolonged disease and none was ill or taking any drug at the time of the study. Blood was taken from all the subjects during the same period of time (6 months). The sera were stored at −70°C until assay.

*Laboratory tests*: Serological testing for anti-HCV was performed using a second generation commercial ELISA (Ortho Diagnostic Systems, Raritan, NJ, USA)

© 1994 Rapid Communications of Oxford Ltd according to the manufacturer's instructions. Anti-HCV reactive samples were confirmed using a second generation anti-HCV recombinant immunoblot assay (RIBA II, Chiron Corporation, Emeryville, CA, USA). In this test the nitrocellulose strip contains four viral antigens, three of the non-structural regions (5–1–1; C-100; C-33c) and one structural peptide (c 22–3). Patients were defined positive when specimens reacted with at least two of the bands, indeterminate when specimens reacted with one band only and negative when there was no reaction. Hepatitis B core antigens were tested using the Abbott RIA kit (Abbott Laboratories, N. Chicago, IL, USA).

The main biochemical parameters of liver function were assayed using commercially available kits.

Liver biopsy specimens were obtained percutaneously with a Menghini needle and CAH was defined according to the criteria of De Groote *et al.*[11]

*Quantification of soluble molecules:* An enzyme immunoassay test has been used to quantify sIL-2R, sCD4 and sCD8. The assays were performed with commercially available kits purchased from Cellfree (T Cell Sciences, Inc., Cambridge, MA, USA). All tests were performed according to the manufacturer's instructions. Detection limits in the authors' laboratory for sIL-2R, sCD4 and sCD8 were 50 U/ml, 12 U/ml and 50 U/ml, respectively.

*Statistical analysis*: All data are expressed as means ± S.D. Correlations were calculated by linear regression. The values for the different groups were compared using Student's *t*-test.

### Results

Patients with chronic type C hepatitis have significantly higher levels of sIL-2R than do normal controls (Table 1). For the soluble forms of CD4 and CD8, sCD8 levels were significantly increased in patients, whereas for sCD4 no significant difference was observed between the two groups, as shown in Table 1. It is interesting to note that in spite of the simultaneous increase of both sIL-2R and sCD8, no correlation was demonstrated between the levels of these two molecules ($r = 0.25$; $p$ = n.s.). In addition, no significant correlation between serum levels of sIL-2R or sCD8 and those of alanine amino transferase (ALT) or other markers of liver disease was observed (data not shown).

**Table 1.** Serum levels (mean ± S.D.) of soluble IL-2R, CD4 and CD8 in 51 normal controls and 33 chronic type C hepatitis patients

| | Serum levels of glycoprotein | |
|---|---|---|
| Glycoprotein | Healthy subjects | Patients |
| sIL-2R | 497 ± 786[a] | 1511 ± 972[b] |
| sCD4 | 30 ± 15[c] | 34 ± 19[d] |
| sCD8 | 389 ± 147[e] | 941 ± 384[f] |

*p* values for: a *vs.* b, $2.77^{-6}$; c *vs.* d, $1.82^{-14}$; e *vs.* f, n.s.

**Table 2.** Serum levels (mean ± S.D.) of soluble IL-2R and CD8 in 22 patients at day 0 after 6 months

| | Serum levels of glycoprotein | |
|---|---|---|
| Glycoprotein | At day 0 | After 6 months |
| sIL-2R | 1233 ± 506[a] | 1275 ± 473[b] |
| sCD8 | 900 ± 357[c] | 879 ± 268[d] |

*p* values for: a *vs.* b, n.s.; c *vs.* d, n.s.

In a small number of patients, available for a second observation after 6 months, the sera showed mean levels of these soluble molecules almost identical to those of the first observation (Table 2).

### Discussion

The present results demonstrate that in patients affected by chronic type C hepatitis there is a significant increase of sIL-2R and sCD8 but not of sCD4. This increase is present during the whole course of the disease as demonstrated by the fact that a second assay of these soluble molecules on the sera of the same patients 6 months later showed no significant changes.

Serum sIL-2R has been measured in patients affected by several liver diseases. All patients with acute type B hepatitis presented levels significantly higher than that of normal controls or of patients with chronic type B hepatitis. Serial follow-up showed that serum levels of sIL-2R tended to return to normal 2–4 months after onset of acute hepatitis together with the ALT normalization. Patients with chronic type B hepatitis also had significantly higher levels of sIL-2R that varied considerably with liver flogosis, i.e. significantly lower levels were detected in patients with chronic infection who had no evidence of active liver disease. In chronic infection, in response to therapy with prednisone and/or interferon, serum sIL-2R fell significantly and a significant correlation between serum sIL-2R and ALT levels has been observed. High sIL-2R levels have also been observed during hepatitis A infection, while lower values have been seen during acute hepatitis C infection.

Soluble IL-2R has also been measured in the serum of patients with liver cirrhosis, patients with obstructive jaundice and patients with alcoholic liver disease without evidence of cirrhosis. In patients with cirrhosis and obstructive jaundice sIL-2R was significantly increased compared with healthy subjects. No difference was found between patients with cirrhosis due to alcohol abuse and chronic hepatitis B.[10–12] In obstructive jaundice, sIL-2R correlated with alkaline phosphatase as a marker of cholestasis.[13]

Concerning serum sCD8 levels, this glycoprotein was increased in patients with cirrhosis but not in patients with obstructive jaundice, compared with healthy subjects and patients with unrelated diseases. No difference was found between patients with cirrhosis due to alcohol abuse and chronic hepatitis B.[12–15]

On the whole these data show that in spite of the apparent well-known depressed cellular immune response both in liver cirrhosis and obstructive jaundice[14,15] there is a general activation of the immune system. The same is true for type C chronic hepatitis (present results). However, in other liver diseases there may be a correlation between the increase of soluble factors and the signs of liver impairment (for instance in chronic type B hepatitis there is a strong correlation between serum sIL-2R levels and ALT, and in jaundice there is a correlation between sIL-2R levels and alkaline phosphatase). This does not occur in CAH. The lack of correlation strengthens the suggestion that the activation of the immune system does not play a role in the pathogenesis of chronic type C hepatitis that is due almost exclusively to a cytopathic effect of virus.[7–10]

### References

1. Caruso C, Candore G, Cigna D, Colucci AT, Modica MA. Biological significance of soluble IL-2 receptor. *Mediators of Inflammation* 1993; **2**: 3–21.
2. Tomkinson BE, Brown MC, Ip SH, Carrabis S, Sullivan JL. Soluble CD8 during T cell activation. *J Immunol* 1989; **142**: 2230–2236.
3. Symons JA, McCulloch JF, Wood NC, Duff GW. Soluble CD4 in patients with rheumatoid arthritis and osteoarthritis. *Clin Immunol Immunopathol* 1991; **60**: 72–82.
4. Symons JA, Wood NC, Di Giovine FS, Duff GW. Soluble CD8 in patients with rheumatic diseases. *Clin Exp Immunol* 1990; **80**: 354–359.
5. Zielinski CC, Pesau B, Muller Ch. Soluble interleukin-2 receptor and soluble CD8 antigen in active rheumatoid arthritis. *Clin Immunol Immunopathol* 1990; **57**: 74–82.
6. Bresson-Hadni S, Monnot-Jacquard B, Racadot E, Lenys D, Miguet JP, Vuitton DA. Soluble IL-2-receptor and CD8 in the serum and the periparasatic granuloma of patients with alveolar echinococcosis. *Eur Cytokine Netw* 1991; **2**: 339–344.
7. Prince AM, Brotman B, Huima T, Pascual D, Jaffery M, Inchuspè G. Immunity in hepatitis C infection. *J Infect Dis* 1992; **165**: 438–443.
8. Czaja AJ. Chronic hepatitis C virus infection—a disease in waiting? *N Engl J Med* 1992; **327**: 1949–1950.
9. Farci P, Alter HJ, Govindarajan S, *et al.* Lack of protective immunity against reinfection with hepatitis C virus. *Science* 1992; **258**: 135–140.
10. Botarelli P, Brunetto MR, Minutello MA, *et al.* T-lymphocyte response to hepatitis C virus in different clinical courses of infection. *Gastroenterology* 1993; **104**: 580–587.
11. De Groote J, Desmet VJ, Gedik P, *et al.* A classification of chronic hepatitis. *Lancet* 1968; **ii**: 628–631.
12. Yamaguchi S, Onji M, Ohta Y. Increased serum soluble interleukin-2 receptor levels in patients with viral liver disease. *Hepatogastroenterology* 1988; **35**: 245–248.
13. Chu CM, Liaw YF. Serum levels of soluble Tac peptide in acute and chronic hepatitis B virus infection. *Clin Immunol Immunopathol* 1989; **53**: 52–58.
14. Leung NW, Leung JC, Tam JS, *et al.* Effects of alpha-interferon and prednisone on serum soluble interleukin-2 receptor (sIL-2R) in chronic hepatitis B infection. *J Gastroenterol* 1992; **87**: 113–117.
15. Wagner F, Assemi C, Lersch C, Hart R, Classen N. Soluble interlerukin-2 receptor and soluble CD8 in liver cirrhosis and obstructive jaundice. *Clin Exp Immunol* 1990; **82**: 344–349.
16. Wejstal R, Norkrans G, Weiland O, *et al.* Lymphocyte subsets and $\beta_2$-microglobulin expression in chronic hepatitis C-non-A, non-B. Effects of interferon-alpha treatment. *Clin Exp Immunol* 1992; **87**: 340–345.

ACKNOWLEDGEMENTS. This work was supported by a grant from the Ministero dell'Università e della Ricerca Scientifica e Tecnologica to C. C.

**Received 19 January 1994; accepted 14 February 1994**
